# A New Method to Evaluate Pressure Distribution Using a 3D-Printed C2-C3 Cervical Spine Model with an Embedded Sensor Array

**DOI:** 10.3390/s23239547

**Published:** 2023-12-01

**Authors:** Maohua Lin, Rudy Paul, Xinqin Liao, James Doulgeris, Emma Lilly Menzer, Utpal Kanti Dhar, Chi-Tay Tsai, Frank D. Vrionis

**Affiliations:** 1 Department of Ocean and Mechanical Engineering, Florida Atlantic University, Boca Raton, FL 33431, USA; mlin2014@fau.edu (M.L.); paulr2017@fau.edu (R.P.); udhar2020@fau.edu (U.K.D.); tsaict@fau.edu (C.-T.T.); 2 Department of Electronic Science, Xiamen University, Xiamen 361005, China; liaoxinqin@xmu.edu.cn; 3 Department of Biological Sciences, Florida Atlantic University, Boca Raton, FL 33431, USA; 4 Department of Neurosurgery, Marcus Neuroscience Institute, Boca Raton Regional Hospital, Boca Raton, FL 33486, USA

**Keywords:** sensor array, finite element analysis, 3D print, cervical spine, ACDF

## Abstract

Cervical degenerative disc diseases such as myelopathy and radiculopathy often require conventional treatments like artificial cervical disc replacement or anterior cervical discectomy and fusion (ACDF). When designing a medical device, like the stand-alone cage, there are many design inputs to consider. However, the precise biomechanics of the force between the vertebrae and implanted devices under certain conditions require further investigation. In this study, a new method was developed to evaluate the pressure between the vertebrae and implanted devices by embedding a sensor array into a 3D-printed C2-C3 cervical spine. The 3D-printed cervical spine model was subjected to a range of axial loads while under flexion, extension, bending and compression conditions. Cables were used for the application of a preload and a robotic arm was used to recreate the natural spine motions (flexion, extension, and bending). To verify and predict the total pressure between the vertebrae and the implanted devices, a 3D finite element (FE) numerical mathematical model was developed. A preload was represented by applying 22 N of force on each of the anterior tubercles for the C2 vertebra. The results of this study suggest that the sensor is useful in identifying static pressure. The pressure with the robot arm was verified from the FE results under all conditions. This study indicates that the sensor array has promising potential to reduce the trial and error with implants for various surgical procedures, including multi-level artificial cervical disk replacement and ACDF, which may help clinicians to reduce pain, suffering, and costly follow-up procedures.

## 1. Introduction

For some people with degenerative cervical spine disorders, adverse symptoms such as myelopathy and radiculopathy may emerge as a result of spinal cord injury and nerve root compression [1]. For such individuals, artificial cervical disc replacement is a common surgical approach. However, various complications, such as adjacent disc degeneration, disc space narrowing, and other mechanical issues, continue to pose significant concerns [2,3]. The reported rates of these issues following artificial cervical disc replacement are quite high, with approximately 15% reported in single-level procedures and about 50% in multi-segment procedures [4,5]. These issues are thought to be caused by increased stress in the adjacent level, which leads to hypermobility and quicker degeneration.

Measuring the pressure distribution of intervertebral discs, both in vivo and in vitro, is critical for evaluating biomechanical stability. Presently, the methodological tools available for in vivo measurements of intervertebral disc pressure are limited. Few human spine studies have measured intervertebral disc pressure. Due to the rising interest in measuring this pressure, in vitro and in vivo studies have been performed on various animal models. Needle-mounted strain gauge pressure sensors have been traditionally applied for measuring the disc pressure both in vitro and in vivo; however, they provide a single pressure measurement as opposed to a comprehensive pressure distribution. A minuscule needle-type pressure sensor has been employed to measure static disc pressure, though overall, tools for evaluating intervertebral pressure distribution are lacking [6,7,8,9,10,11,12,13,14]. Ultimately, surgeons have to select cervical disk implants solely based on diagnostic imaging studies. Rather than relying primarily on imaging and analog measurements, a better understanding of this pressure distribution may aid surgeons in implant selection.

An accurate representation of the anatomy and biomechanics of a patient’s spine should occur at the outset of treatment [15,16,17,18,19,20,21,22,23]. Traditionally, understanding the biomechanics of a patient’s spine is accomplished through medical imaging. However, a promising alternative method proposes using wearable sensors to acquire these geometric parameters, as their easy and swift integration is advantageous compared to high-cost imaging systems [24,25,26]. Intradiscal pressure measurements can be generated using physical models of a patient’s spine. Various methods have been put forth for creating a sensor array to measure pressure at multiple points across a plane [27,28,29,30]. Although, integrating such a sensor into a model without hindering joint-like flexibility requires flexible sensing technologies.

**Figure 1:**
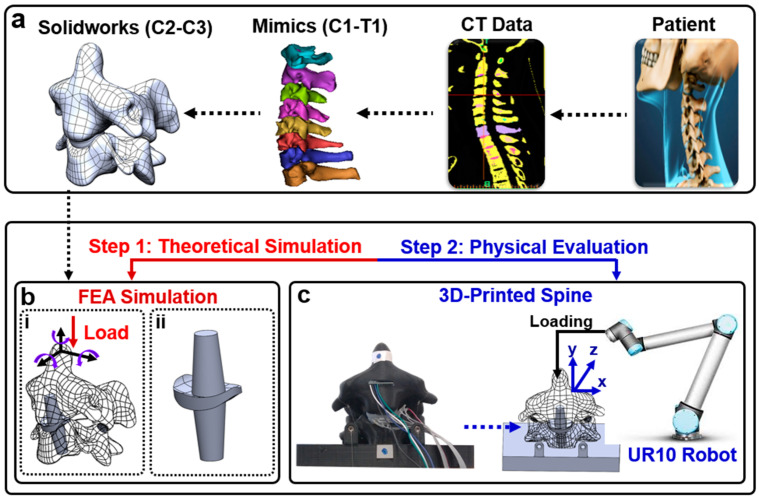
(**a**) Patient CT data are edited in Mimics to create a 3D model that can be edited in Solidworks; (**b**) (**i**) spine with artificial disc replacement will be compared in simulation to both predict the potential for improvement and choose the best possible replacement option; (**ii**) bone graft substitute with implanted pressure sensor; (**c**) the sensorized artificial disc will be embedded into the computer numerical control (CNC)-fabricated spine model. A robotic arm will manipulate the spine model.

In this study, a sensor array was incorporated into a soft silicone replica of the intervertebral disc for a physical model identical to CT data. A 3D model was developed from CT data (Figure 1a) to reproduce the pressure responses with finite elements (Figure 1b) and in a physical model (Figure 1c). Given this methodology’s low cost, non-demanding equipment, and patient-specific approach, it has the potential to guide the management of cervical disc arthroplasty through the acquisition of previously unattainable in vivo biomechanical measurements.

## 2. Materials and Methods

### 2.1. Fabrication

The flexible tactile sensor array employs two main piezoresistive components: velostat and stainless-steel thread. Velostat, a composite film of polyethylene and carbon black, undergoes conductivity changes under pressure. Likewise, the stainless-steel thread also exhibits increased conductivity when force is applied. Pressing on velostat reduces the distance between carbon black particles, increasing contact points and altering conductivity in the affected film area. Similarly, applying force to the stainless-steel thread boosts conductivity by increasing contact points within the thread. By arranging these threads in a grid connected to the conductive film, a sensor array is formed to measure pressure distribution across the grid surface.

The sensor is constructed by assembling seven layers, including an outer layer, an adhesive layer, longitudinal wires, a conductive layer, transverse wires, another adhesive layer, and a second outer layer. The outer layers use plastic wrap (MRP Corp, Philadelphia, PA, USA), and the adhesive layer consists of 3M double-sided adhesive (3M™ Adhesive Transfer Tape 468MP, St. Paul, MN, USA). Stainless steel yarn forms the wires (Stainless Thin Conductive Thread—2 ply, Adafruit Industries LLC, New York, NY, USA), and the conductive layer is velostat, a pressure-sensitive and conductive sheet (Velostat 1361, Adafruit Industries LLC). The assembly process involves cutting rectangles of velostat to match the sensor’s size and cutting plastic wrap and adhesive into a 2 cm × 1.5 cm rectangle. Wires are placed in a 3D-printed holster to maintain an even spacing of 0.2 cm for longitudinal wires and 0.3 cm for transverse wires. The wires are lightly pressed onto one adhesive layer, and the assembly is placed on top of the conductive layer. After trimming the adhesive to match the conductive layer, the protective layer is removed to expose the other side of the adhesive. The outer layer is wrapped around a finger, rolled onto the adhesive to prevent air pockets, and the process is repeated for the other side of the conductive layer with transverse wires. Longitudinal wires are soldered and encapsulated in heat-shrink tubing separately, while all wiring along the fingers are encapsulated together in heat-shrink tubing (Electriduct, 3.18 mm 3:1 polyolefin tubing). The horizontal channels are soldered after the Dragon Skin 30 cures to prevent unnecessary strain on the steel thread during fabrication. The ultimate dimensions of the sensor are 1.5 cm × 1 cm.

**Figure 2:**
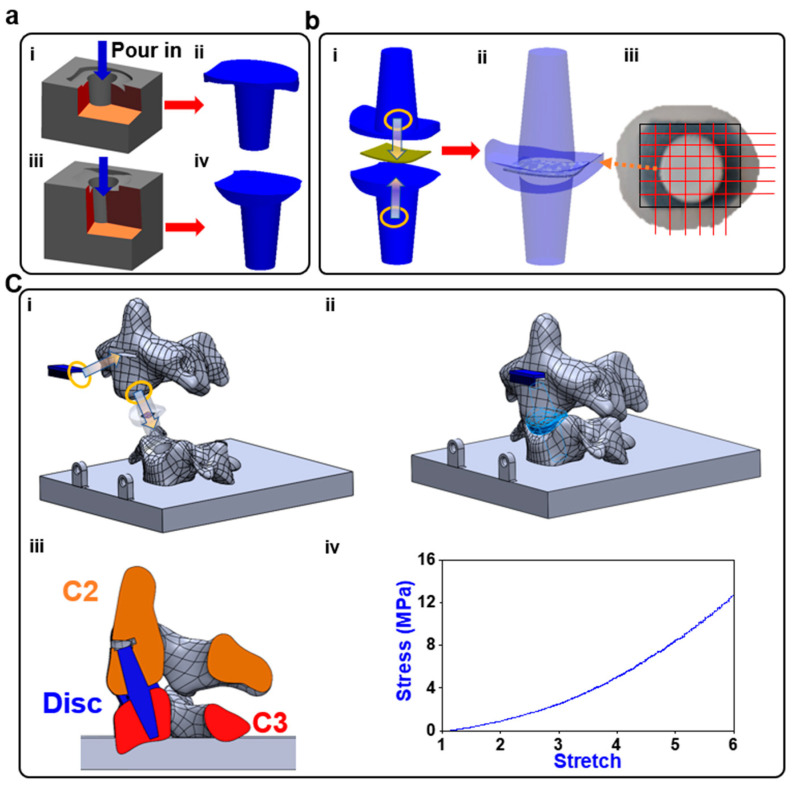
Fabrication process: (**a**) (**i**–**iv**) two molds are printed on the two halves of the disc and both are filled with DS; (**b**) (**i**) the sensor is centered in between the two halves; (**ii**) the halves are sealed together using DS; (**iii**) the alignment of the sensor is verified, and then the wiring is soldered on; (**c**) (**i**) the disc and inertial measurement unit (IMU) sensor are installed into the corresponding locations that are filled with DS; (**ii**) the DS cures leaving behind an anchor to which the disc bonded; (**iii**) the cross-section view of the model; (**iv**) the stress–stretch performance of the Dragon Skin 30.

A computerized tomographic (CT) scan of a patient’s cervical spine was imported into Solidworks™ (Dassault systems, Velizy-Villacoublay, France) and edited to allow the incorporation of sensors and proper contact behavior during physical testing. The molds were made from the edited model’s disc geometry and cast in Dragon Skin 30 (DS), (Smooth-On, Macungie, PA, USA), which was selected as the medium due to its low delamination and high flexibility DS (Figure 2a). The sensor array was placed between the two halves of the disc, and the halves were combined using DS (Figure 2b(i,iii)). The centering of the 6 × 6 sensor array was checked (Figure 2b(iii)) and the CNT wires were soldered to an appliance wiring material (AWM) 2 × 6 female socket. Each vertebra was printed with an open cavity. The disc was inserted into the 2 vertebral segments, C2 and C3, and the cavities were filled with DS so that the cured DS would combine with the disc, so that the disc was fixed in place. The anterior face of the C2 vertebral body was filled with DS to anchor the IMU sensor within it (Figure 2c). 

The vertebra sections, the essential components of our study, were meticulously crafted using state-of-the-art 3D printing technology with PLA (polylactic acid) material. The decision to employ 3D printing not only ensured precision in replicating anatomical structures but also allowed for customization to achieve the desired dimensions and geometries. Importantly, the infill percentage for the 3D-printed vertebra sections was set at 20%, providing substantial structural integrity and robustness during the application of small forces. This meticulous approach in material selection and printing parameters enhances the fidelity of our experimental setup, providing a reliable foundation for the subsequent finite element analysis and pressure distribution assessments in the context of cervical spine biomechanics.

### 2.2. FEA Simulation

**Figure 3:**
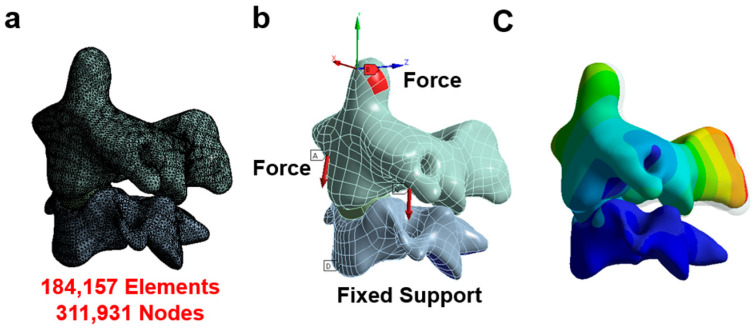
Finite element model. (**a**) The mesh of the model; (**b**) the boundary and loading condition in each situation (A indicates force and D indicates fixed support); (**c**) the displacement contour.

The CT data were converted into a refined CAD model using Mimics [19,31] and Hypermesh [32,33,34]. The model was edited in Solidworks and exported to ANSYS workbench 19.1 for analysis [20,35,36]. This original model was used in our previously published paper and the results were consistent with the range of other FEA models and in vivo studies for all movements [37,38,39,40]. The conditions of the physical model dictated the setup of the finite element analyses (FEAs). The individualized FEA was used to characterize the outputs of the sensor as pressure distribution in the given scenario. In Figure 3, the boundary conditions are depicted, highlighting the points of load application in each condition. The vertebra was divided into two components: the cortical bone and the cancellous bone. The cortex was represented using shell elements, with a set thickness of 0.2 mm. Meanwhile, the cancellous bone was simulated using solid elements (tetrahedral elements). A convergence analysis was conducted to ensure the adequacy of mesh sizing, achieving a convergence criterion of less than 5%. The results from the convergence test demonstrated that the solution did not significantly alter with mesh refinement, as illustrated in Figure 3a. Notably, the solution showed minimal changes even with mesh refinement, maintaining consistency with 311,931 nodes and 184,157 elements. 

The Young’s modulus for polylactic acid (PLA) was reported to be 3.5 GPa, with a Poisson ratio of 0.36 [41]. The stress–stretch performance of the Dragon Skin 30 was shown in Figure 2c. High flexibility was the sensor’s primary requirement, while thickness was considered negligible; hence, the properties of paper were used in the simulation (5.1 GPa Young’s modulus and 0.306 Poisson ratio [42,43]). The facet joint was regarded as an empty gap and the surfaces were assigned a frictionless contact. The loading conditions created on the UR-10 were replicated using a compressive force on the tip of C2 to simulate flexion, extension, right or left lateral bending, or axial compression. The preload was represented by applying 22 N of force on each of the anterior tubercles for the C2 vertebra. The lower surface of the C3 vertebra of the model was constrained. Facet joint contact problems [44] were solved nonlinearly by surface-to-surface contact elements, and the frictional coefficient was assumed as 0.42, the coefficient for PLA. The surface contacts between the discs’ anchors were set as bonded, and the contact between the disc and the vertebra was set as rough. The contact between the disc and the embedded sensor was set as bonded.

### 2.3. Physical Evaluation of Pressure Sensor Array and Range of Motion

Before implementation in the artificial cadaver test, benchtop testing was performed to evaluate the sensor’s performance. The sensor was secured to a load cell and the replica of the patient’s cervical spine was 3D-printed using PLA; the stress in the vertebra was irrelevant in the study and the difference in strength between the vertebra and disc were great enough to make the specific properties negligible. To measure the pressure between the artificial disc and vertebra at multiple contact points, a new sensor array was customized to fit the disc. The pressure sensor array was centered and encased within the disc. An adaptor for the robotic arm–spine interface was designed to apply a compressive force to the tip of the vertebra. The ROM of the vertebra was controlled using the UR-10 and monitored using an IMU sensor. The vertebra was deflected in each motion until the sensor output a noticeable response. A preload cable was attached to each of the anterior tubercles of the C2 vertebra. The preload cable was passed through the transverse process of the C3 vertebra and off the side of the printed plate where 5 lb weights were attached and hung from the ends. The C3 vertebra was printed within a rectangular plate and the plate was attached to a load cell that was clamped to the table.

### 2.4. Data Acquisition System

**Figure 4:**
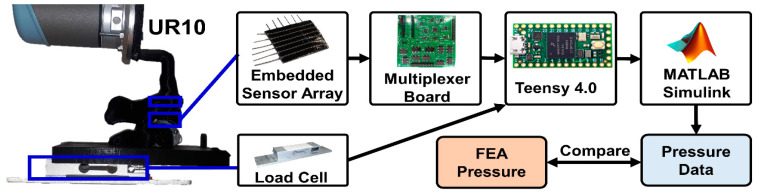
Control system: the UR-10 applied loads to the spine replica, which was equipped with an IMU, a pressure sensor array, and a load cell. The sensor array’s 36 taxels were sampled with a multiplexor board. All three sensors’ outputs were sent through a Teensy 4.0 to be recorded in Simulink.

The setup of the physical model for testing and data collection is shown in Figure 4. The embedded sensor array’s electrical resistance corresponds to the force applied at each sensor node. Each row and column of the sensor was connected to a multiplexer board. The board functioned by multiplexing the rows and columns of the sensor array and activating a single pair at a time to be used as a resistor alongside a fixed 24 kΩ resister, which was amplified and low-pass filtered. The sampling of the sensor voltages was managed using a Teensy that was programmed to cycle through all 36 taxels, which were subsequently published into a local robot operating system (ROS) network. The load cell was connected to an operational amplifier, and the output of the OP-AMP was also published to a local ROS network using a Teensy. In Simulink 2023 (The Mathworks, Natick, MA, USA), all the data were collected from the ROS network and passed through a 5 Hz digital low-pass filter.

## 3. Results

### 3.1. Sensor Properties

**Figure 5:**
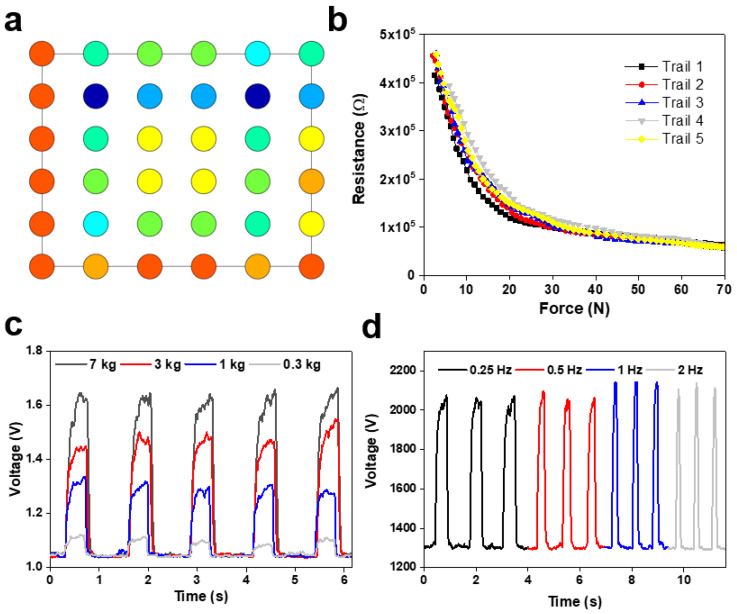
Characteristics of the developed flexible pressure sensor array: (**a**) presentation of the 36 taxels as a color map that is proportional to pressure across the surface of the sensor (different color means different value of the sensor voltage); (**b**) chart displaying the sensitivity and operating range of the sensor; (**c**) the response of a single taxel at the application of varying force; (**d**) response of a single taxel at varying frequencies of application.

In this work, we developed and calibrated a soft tactile sensor array that can resolve the size and location of the normal load applied to its surface. The sensor was tested in a shear force application and proved ineffective. The taxel outputs of the sensor were successfully adapted into a pressure distribution map (Figure 5a). The resistance of the taxels changed in response to external forces (Figure 5b). The sensor performed well from 0.3 to 7 kg but suffered greater delays in peak time as the pressure increased (Figure 5c). The delay of the sensor also meant that the signal retention suffered greatly as the frequency of cycles increased (Figure 5d).

### 3.2. Flexion Application

**Figure 6:**
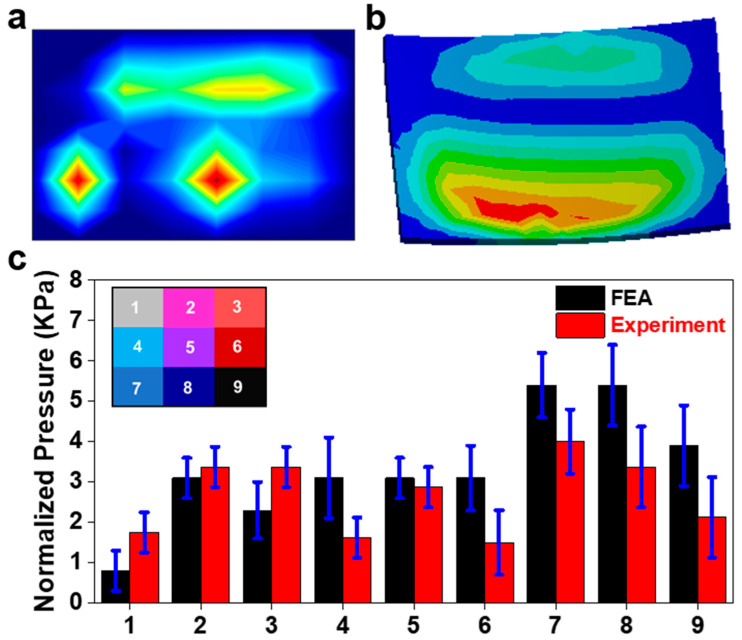
Flexion application. (**a**) Pressure field from sensor array during the physical experiment (red color means higher value and blue color means lower value); (**b**) the pressure field from the sensor array FE model; (**c**) comparison of normalized pressure between FEA and experiment in nine areas with a map of their locations on the sensor.

Figure 6a shows the pressure field of the sensor array obtained from Simulink during the recreation of flexion. The pressure distribution on the sensor from the FEA is displayed in Figure 6b. The assignment of nine areas across the surface of the sensor and the normalized pressure of each area is displayed in Figure 6c. The peak pressure is about 5.5 kPa near area eight of the sensor. Significant pressure appears in all nine areas, as expected for a case of compression from a neutral position. Figure 6c compares the pressures in each area for both the FEA and physical model. Areas one and four showed the greatest discrepancy between the FE analysis and the experiment but were still under the range. The pressure concentrations on the anterior area eight of the sensor are much more dispersed in the FE results, which may account for the larger discrepancies in these areas. There were some tactile peaks in areas three and four. The change in preload had a minimal effect on the sensor response.

### 3.3. Extension Application

**Figure 7:**
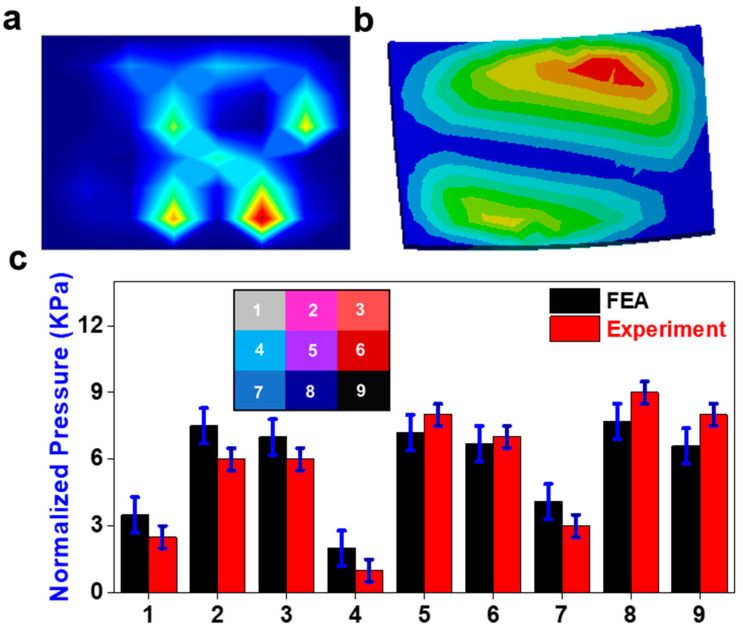
Extension application. (**a**) Pressure field from sensor array during the physical experiment (red color means higher value and blue color means lower value); (**b**) the pressure field from the sensor array FE model; (**c**) comparison of normalized pressure between FEA and experiment in nine areas with a map of their locations on the sensor.

The sensor response was much less dispersed in the posterior region than during extension (Figure 7a,b), though the actual pressure amount was still greater. In the FEA, the pressure contours of extension shared the same main pressure concentrations as during flexion. The pressure experienced in this condition was much greater than it was during flexion (Figure 7c). The two pressure regions are similar, but the sensor showed greater pressure in the anterior region. There is no significant average difference between the physical sensor and the FE results. Area nine showed the greatest discrepancy between FEs and the experiment with an error of 0.2 kPa. Once again, the pressure distribution is significantly more gradual in the FE results when compared to the sensor output.

### 3.4. Bending Application

**Figure 8:**
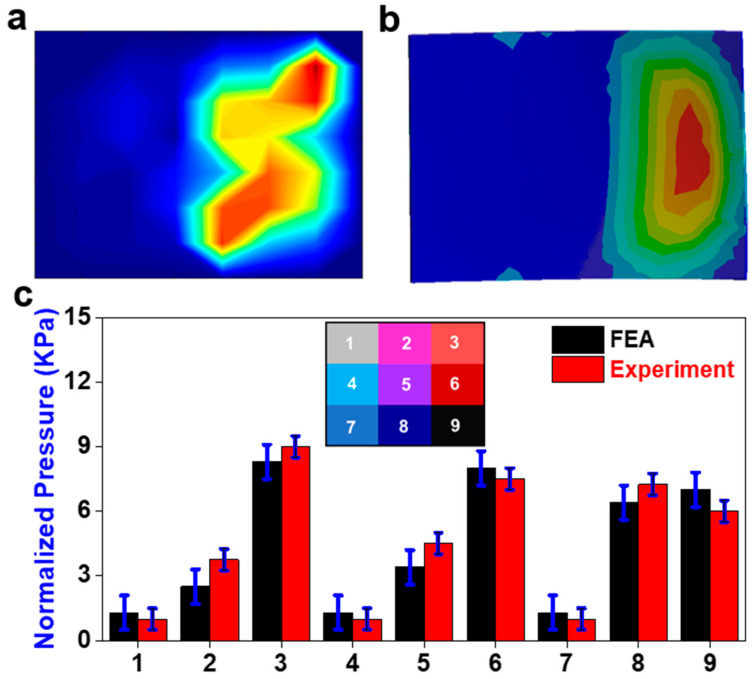
Bending application. (**a**) Pressure field from sensor array during the physical experiment (red color means higher value and blue color means lower value); (**b**) the pressure field from the sensor array FE model; (**c**) comparison of normalized pressure between FEA and experiment in nine areas with a map of their locations on the sensor.

The bending application seemed to have the most correlation between the experimental and FE results. There is no significant difference between the physical sensor and the FE results. The physical sensor had a steeper pressure gradient (Figure 8a), yet the pressure seems to be more concentrated on the right side of the sensor in the FE results (Figure 8b). The difference in location of the pressure gradient is displayed in the consistently higher-pressure outputs in areas two, five, and eight. The steepness of the physical sensor’s pressure gradient can be seen in the contrast of outputs from areas one to two, four to five, and seven to eight (Figure 8c). The change in preload had a minimal effect on the sensor response. 

### 3.5. Compression Application

**Figure 9:**
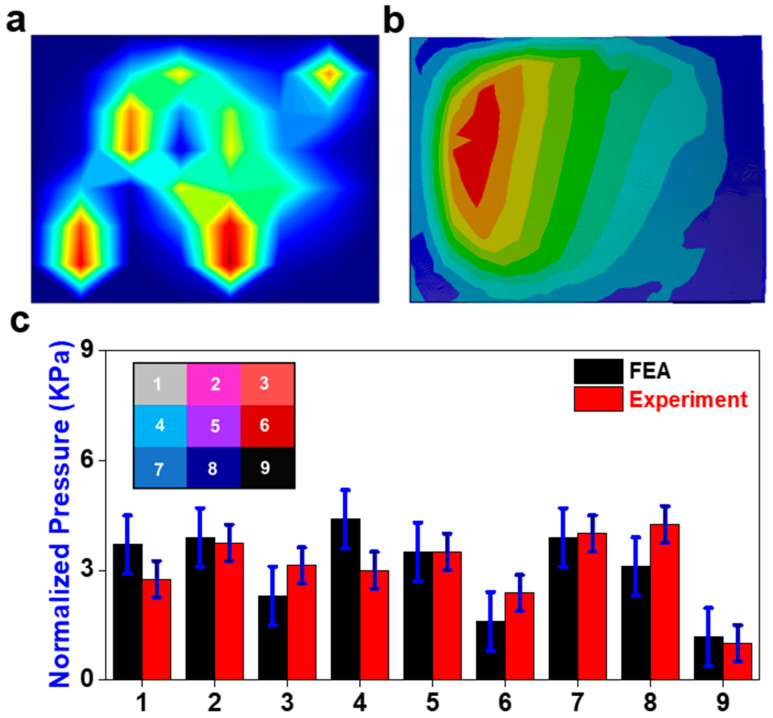
Compression application. (**a**) Pressure field from sensor array during the physical experiment (red color means higher value and blue color means lower value); (**b**) the pressure field from the sensor array FE model; (**c**) comparison of normalized pressure between FEA and experiment in nine areas with a map of their locations on the sensor.

Compression application shows the pressure distribution across the sensor was similar between both results (Figure 9). However, the physical sensor had several high-pressure concentrations in comparison to a single continuous one (Figure 9a). In addition, there was a high-pressure concentration in area eight that was not present in the FE results (Figure 9b). There is no significant difference between the physical sensor and the FE results. Area four showed the greatest discrepancy between the FE and experiment results (Figure 9c). The change in preload had a minimal effect on the sensor response. 

## 4. Discussion

Our study significantly contributes to the cervical spine biomechanics literature by providing unique insights into pressure distribution during flexion, extension, and bending actions. The findings align with existing biomechanical knowledge but offer nuanced perspectives, particularly in optimizing cage parameters based on pressure distribution. The incorporation of novel replicas and advanced analytical tools enhances its methodological depth, advancing the discourse on cervical spine surgery and biomechanical stability.

In the flexion condition, significant pressure is present across the whole surface, with an area of high pressure biased towards the right side in the anterior region of the disc. The pressure is distributed evenly across the disc, radiating from the expected central loading point. Thus, it can be predicted that a slight reduction in the existing disc height in the right anterior region to induce a laterally balanced lordosis is sufficient to handle and distribute the load evenly across the surface. During implant placement, the location of the cage would be centralized using the pressure distribution as a guide. Stress is distributed across the surface of the cage rather than in concentrations at the edges of the cage; this indicates a lower likelihood of cage subsidence, which can decrease the neuroforamen space, causing cervicalgia, cervical radiculopathy, and biomechanical instability [45].

During extension, significant pressure is present across the whole surface, with an area of high pressure biased toward the left side in the posterior region of the disc. Therefore, it can be predicted that a reduction in the existing disc height in the left posterior region to induce a laterally balanced lordosis would likely be sufficient to handle the load and distribute it evenly across the surface. The determination of the width parameter of a cage would simply be designed so that, at a set location, the disc would exceed a predetermined amount from the high-stress locations during both flexion and extension.

The area of high pressure is distributed quite evenly across the left side of the disc during bending actions. With this, it can be predicted that the height and lordosis of the current disc are sufficient to handle and distribute the load evenly across the surface. The determination of the length parameter of a cage would simply be designed so that, at a set location, the disc would exceed a predetermined amount from the high-stress locations of the left and right bending.

Using this novel replica with FEA provides enough detailed information to offer insights and analyses. Similar systems are already in place for optimizing spine surgeries using 3D models and FEA [35]. Understanding how a given cage responds under different moments and loadings can aid in designing the optimal cage for a patient’s specific geometries and daily activities. Furthermore, the retention of the patient’s natural range of motion (ROM) is essential. Limiting the patient’s ROM will have a noticeable effect and decrease their quality of life. Too much freedom in ROM can lead to cervical misalignment that may cause radiculopathy and increase the onset of disc degeneration and injury in adjacent levels [3]. Each patient has a unique cervical geometry, which means that there is room for optimizing cage placement to generate the most significant reduction in stress concentrations. A better distribution of load will decrease the risk of cage subsidence and increase the overall biomechanical stability.

The development of the presented work may be useful for the development of wholistic cadaver models that are highly accurate yet completely fabricated. Such a model would provide greater context for spine biomechanics through the integration of soft, flexible sensors such as the one used in the presented model. In addition, the usage of CT reconstruction would produce models that are of far greater use to the individual patient in comparison to the cadaver tests that are currently performed, which give a more generalized result. The current experimental results are applicable to all models, but do not give specific results that would be helpful in picking the best surgical intervention for a given patient. To be used to this capacity, however, the model must be made much more robust and be proven effective.

This study presents a novel methodology for the improvement of cage designs. In its current state, many limitations exist that hinder its feasibility as an improvement of cervical spine surgical hardware. If these issues are properly addressed, this technique has the potential to revolutionize the way in which cervical spine surgery is handled. The use of a new model for clinical applications requires that it be verified and validated with existing in vitro and FE data. In future renditions, a verified FE model should be prioritized to legitimize the sensor’s capability at representing pressure distribution.

Regarding the sensor’s performance, there is potential for improvement in its spatial resolution that would further enhance the design of cages [46,47]. This would most likely fix the steep pressure gradients that were present. This issue did not sufficiently explain the discontinuities in sensor signals that appeared as a continuous high-pressure concentration in the FE model. If a similar sensor is used for future renditions, individual calibrations of each taxel may be necessary to ensure that the pressure-to-voltage relationship in at each point is the same. The sensor also had drastically different outputs for different conditions, ranging from 1 kPa all the way to 10 kPa; this difference, however, may be a result of high friction at the facet joints, which reduces the ability to properly apply loads to the disc in certain motions.

The sensor had inconsistent results in its transient response, which did not affect the current application, but would be helpful in the determination of quick loading, trauma/fracture scenarios. Some discrepancy was also present between the sensor results and the FEA, but this is believed to be due primarily to the oversimplification of the physical model. A better control scheme for the UR-10 would more accurately represent traditional cadaver tests and, as such, could be modeled using the FE method with more standard loading conditions. The integration of the actual cages/grafts would also be the ideal way to perform the physical tests, so it may be worth it to investigate the integration of the sensor within the devices or at the interface between the device and endplates. Though not necessary for effective use, the implementation of artificial ligaments, muscles, and nerves may be useful in the study of nerve radiculopathy and in patient-specific surgical practice. 

While Dragon Skin 30 is commonly employed as a surrogate for biological tissues, comparing the Young’s modulus values to those of the real intervertebral disc in the cervical spine requires a nuanced quantitative assessment. The Young’s modulus of Dragon Skin 30 typically falls within the range of 200 kPa to 300 kPa. In contrast, the Young’s modulus of an actual intervertebral disc in the cervical spine can vary considerably, generally ranging from 10 MPa to 25 MPa. This substantial difference underscores the inherent challenge of precisely replicating the mechanical properties of natural tissues using synthetic materials. It is crucial to recognize that Dragon Skin 30 provides an approximation rather than an exact match to the complex mechanical behavior of real intervertebral discs. The quantitative differences in Young’s modulus highlight the need for careful interpretation when using Dragon Skin 30 as a biomechanical model for cervical spine intervertebral discs.

Although our finite element (FE) model proves instrumental in understanding pressure distribution in cervical spine biomechanics, it does have inherent limitations. One notable limitation is the oversimplification of the physical model, potentially leading to discrepancies between the sensor results and the FE analysis. Furthermore, the model lacks certain dynamic aspects that are crucial for representing real-world scenarios accurately. Despite these limitations, they can be deemed acceptable for several reasons. First, the primary focus of our study is on pressure distribution patterns during various cervical movements, where the simplified model provides valuable insights. Second, the observed discrepancies between the sensor results and FE analysis can be attributed to the inherent challenges in replicating the complexity of physiological conditions. Given the trade-off between model complexity and practical usability, the limitations, while acknowledged, do not significantly compromise the study’s overall contributions to cervical spine biomechanics and surgical applications. The use of simulated paper in our sensor array, while serving as a substrate for tactile sensors, deviates from traditional paper-like properties in soft robotics. The potential limitation arises from the simulated paper’s inextensibility compared to Dragon Skin, impacting the deformability and strain distribution. This difference in elasticity requires careful consideration during result interpretation. To mitigate this, further analysis and calibration are essential to quantify and address any discrepancies introduced by the simulated paper in our experimental setup.

## 5. Conclusions

In this study, a sensor array was designed and manufactured to obtain pressure information between the endplate and the vertebrae. CT data were used to model, and 3D print the C2-C3 cervical vertebrae. A 3D finite element mathematical model was developed to characterize the pressure distribution and ROM. The results show that high sensitivities, fast response, and high stability of the sensor was achieved, and thus significantly reduced the processing difficulty of the coding. The pressure was all calculated from the FEA results in all flexion, extension, bending and compression conditions. These results indicate that the sensor array has broad prospects for the evaluation of the cage design that each patient receives. This will greatly reduce cases of revision surgery, especially in cases of two or more layers of TDR and ACDF. Much of the current methodology for determining the proper cage depends on IVD height. Every patient has unique spinal geometries that are not simply reduced to height, width, and lordosis angle. The adjustment of these with greater specificity, based on numerical and quantifiable information, brings surgeons closer to selecting interbody cages with a full understanding of their postoperative performance.

## Data Availability

The data that support the findings of this study are available on request from the corresponding author.
